# Understanding the patients’ experience in Primary Technology Enhanced Care Home HbA1c Testing (PTEC HAT) programme—a qualitative study

**DOI:** 10.1186/s12875-025-03034-2

**Published:** 2025-10-13

**Authors:** Kah Pieng Ong, Elya Chen, Evonne Oh, Eng Sing Lee, Wern Ee Tang, David Wei Liang Ng, Valerie Teo

**Affiliations:** 1 National Healthcare Group Polyclinics, 1 Mandalay Road, Singapore, 308205 Singapore; 2 MOH Office for Healthcare Transformation (MOHT), Singapore, Singapore

**Keywords:** Diabetes, Tele-monitoring, HbA1c Testing, Point-Of-Care Testing, Patients' Experience

## Abstract

**Background:**

Type 2 Diabetes Mellitus (T2DM) related healthcare expenditure is expected to rise drastically as the incidence of diabetes associated comorbidities increases. It is vital to maintain an optimum glycaemia for patients with diabetes to reduce the risk of diabetes complications. Given the strong predictive value for diabetes complications, HbA1c remains the gold standard to monitor glycaemic control in contemporary clinical practice. The Primary Technology Enhanced Care (PTEC) Home HbA1c Testing (HAT) Programme is a telehealth programme that is intended to empower low-risk patients to test their HbA1c independently at home, supported with tele-monitoring and review through teleconsultation, saving them up to three clinic visits per year. Given the programme’s reliance on active patient involvement, understanding patient experiences within the programme to identify the enablers and barriers of using various PTEC HAT components is important for guiding iterative improvement and informing scale-up strategies.

**Methods:**

A qualitative study approach was used to explore the in-depth perceptions of the patients who were enrolled into the pilot PTEC HAT programme. The non-adoption, abandonment, scale-up, spread, and sustainability (NASSS) framework was used to guide the development of topic guide and the analysis. The emergent results were categorised into the enablers and barriers.

**Results:**

Coaching by healthcare team and access to supporting materials enabled the patients to complete the programme. The proactive reminder for home HbA1c testing by the in-app chatbot, the flexibility to perform the test round the clock, instant result review and the convenience of teleconsultation following home HbA1c test saved time and reduced clinic visits. Patient characteristic which enabled successful participation included a reasonable level of digital literacy, prior experience with health monitoring, absence of needle-related distress and strong intrinsic motivation. HbA1c reagent storage, syncing results via Bluetooth device, the prolonged onboarding process and the time gap between onboarding and first home-based testing were found to be challenging.

**Conclusion:**

PTEC HAT programme was seen as a good alternative to routine clinic care for T2DM. Refinement in the on-boarding process and better support between onboarding and home-based independent testing could improve patient experience and satisfaction.

**Supplementary Information:**

The online version contains supplementary material available at 10.1186/s12875-025-03034-2.

## Introduction

Type 2 Diabetes Mellitus (T2DM) continues to move up the ranks as one of the leading causes of disability and years of life lost, indicating a global transition in disease patterns toward noncommunicable diseases [1]. It is one of the common chronic diseases in Singapore, with the age standardised prevalence rate of 7.9% [2]. Singapore’s multi-ethnic composition, comprising primarily Chinese (74%), Malay (13.5%), and Indian (9%) communities and others (3.4%) [3], contributes to variations in diabetes risk. The prevalence rates of T2DM are notably higher among Malay and Indian populations compared to Chinese. Singapore is projected to have one million individuals with diabetes by 2050. It has been reported that the healthcare expenditure will rise drastically as the future incidence of diabetes related comorbidities increase [4].

In Singapore, primary care is provided by 24 government-subsidised polyclinics and 1,700 general practitioner (GP) clinics. Singapore's healthcare system is organised into three main healthcare clusters, each serving specific geographical regions. Many patients with chronic diseases are being managed by private and public primary healthcare system. NHG Polyclinics is one of the three national public primary health clusters. It is part of the National Healthcare Group, which serves a population of 1.5 million in Singapore’s Central North region. The seven existing polyclinics are Ang Mo Kio Polyclinic, Geylang Polyclinic, Kallang Polyclinic, Hougang Polyclinic, Toa Payoh Polyclinic, Woodlands Polyclinic and Yishun Polyclinic. The NHG Polyclinics is a one stop centre for primary care needs, which provides management of acute conditions, chronic disease management, women’s health and family planning, childhood immunisation and developmental assessment, health promotion/disease prevention, allied health services (dietetic, psychology, physiotherapy, podiatry, medication management, financial counselling, medical social service, lab and diagnostic service) and dental care.

In the public clinics, also known as polyclinics, subsidies play a major role in keeping patient costs manageable, particularly for chronic diseases such as diabetes. Government subsidies for polyclinic consultations and medications substantially reduce out-of-pocket expenses for citizens and permanent residents.

However, studies show that managing multiple chronic diseases, including diabetes, in public primary care settings still incurs significant healthcare costs even after subsidies, primarily due to regular monitoring, clinic visits and medications [5]. Hence, achieving and maintaining glycaemia at the optimal range is crucial for reducing healthcare costs and minimising the risk of diabetes-related complications and cardiovascular events [6–8].

HbA1c results from glycation, a reaction that involves posttranslational modification of haemoglobin A by the nonenzymatic covalent binding of glucose to the N-terminal valine of the β-globin chain [9]. Low intraindividual biological variability, the stability of the analyte and the independence of results to the prandial status were the most pronounced advantages of HbA1c over plasma glucose. Hence, it remains the gold standard test to monitor glycaemic control in clinical practice [10], especially for low risk patients with type 2 diabetes mellitus (T2DM) [11]. The HbA1c test is traditionally performed at the laboratory located within a clinic or healthcare institution. The American Diabetes Association recommends measuring HbA1c at least twice a year in patients who are meeting treatment goals and quarterly in patients whose therapy has changed or have suboptimal glycaemic control, given its strong predictive value for diabetes complications [12].

Telehealth or the use of technology-based virtual platform for health information, prevention, monitoring and medical care has been widely used in the management of diabetes [13–16]. In Singapore, there is a significant level of willingness to take up telehealth among the diabetes patients in polyclinics, though 52.2% of them reported their willingness only after hearing positive reports [17]. Numerous recent qualitative studies have focused on the patients’ perception of using telehealth for T2DM management, as well the factors for supporting primary care physician engagement with patient mobile app for T2DM self-management. The studies have yielded mixed result [18–20]. In recent years, there have been newer approved HbA1c self-test kits available in the market but there is no study describing the deployment of these devices in published literature.

**Fig. 1:**
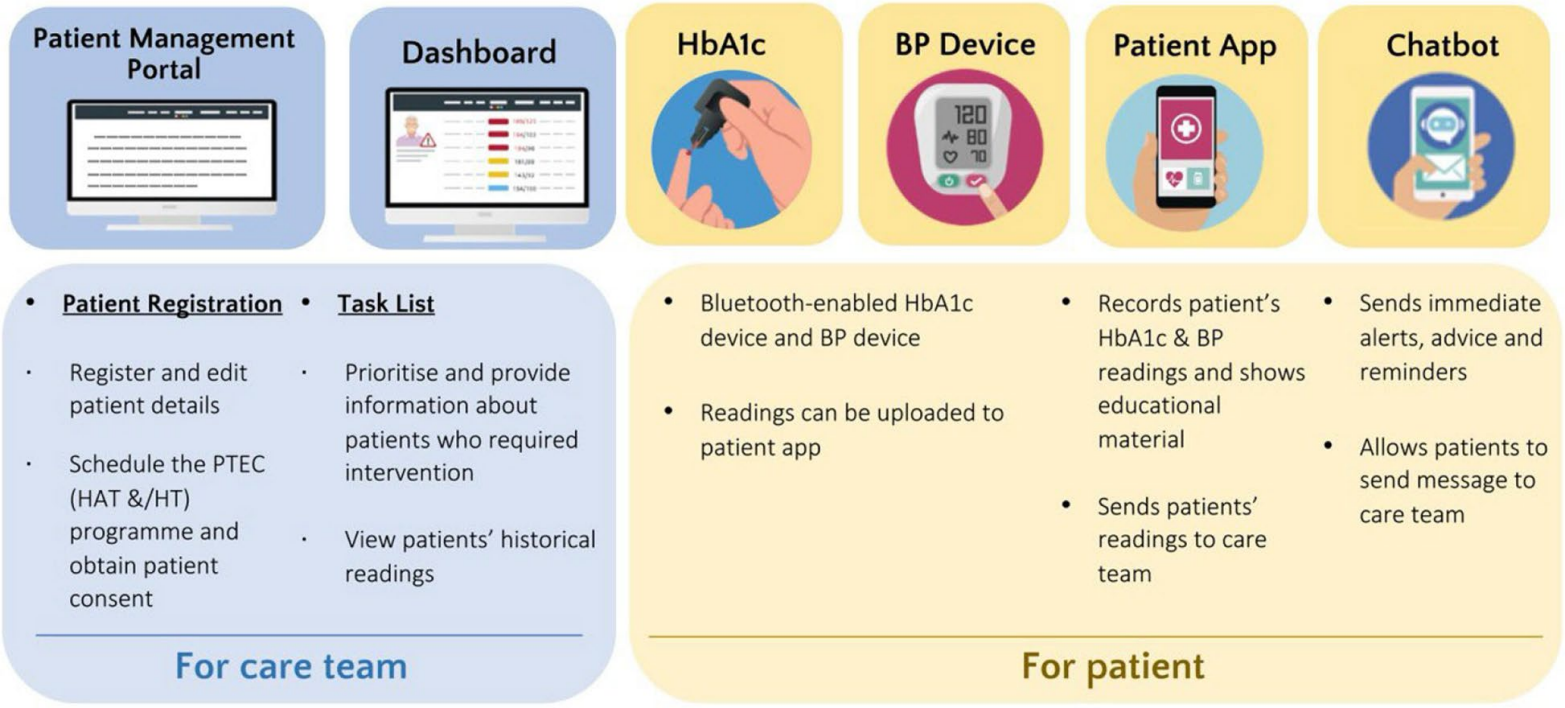
PTEC HAT tech system

PTEC HAT programme is developed by MOHT and collaborated with NHG Polyclinics to implement telehealth care to low-risk patients with T2DM in the community. The programme is sponsored by MOHT and was offered to the patients free of charge during the pilot phase. Low-risk patients are patients who do not require self-blood glucose monitoring (SBGM) or continuous glucose monitoring (CGM), without insulin treatment, without active titration of medication and/or with low risk of hypoglycaemia. The PTEC HAT Tech system, as illustrated in Fig. 1, consists of HbA1c self-test kit, optional blood pressure (BP) machine for patients with coexisting hypertension and a mobile app on the patients’ smartphone (with in-app chatbot and multimedia educational materials access). It is intended to empower low-risk patients with T2DM to test their HbA1c independently, supported with telemonitoring, and maintain their follow-up with the healthcare team through teleconsultations. By participating in PTEC HAT programme, eligible patients will be able to replace their three to six monthly interim paired HbA1c test and physical polyclinic visits with home HbA1c tests and teleconsultations. The frequency of home HbA1c test and teleconsultation will be determined by the reviewing doctor during polyclinic visit following the annual diabetes panel test. This allows patients to save up to three polyclinic visits per year while getting their glycaemic control telemonitored and remotely cared by the healthcare team.

**Fig. 2:**
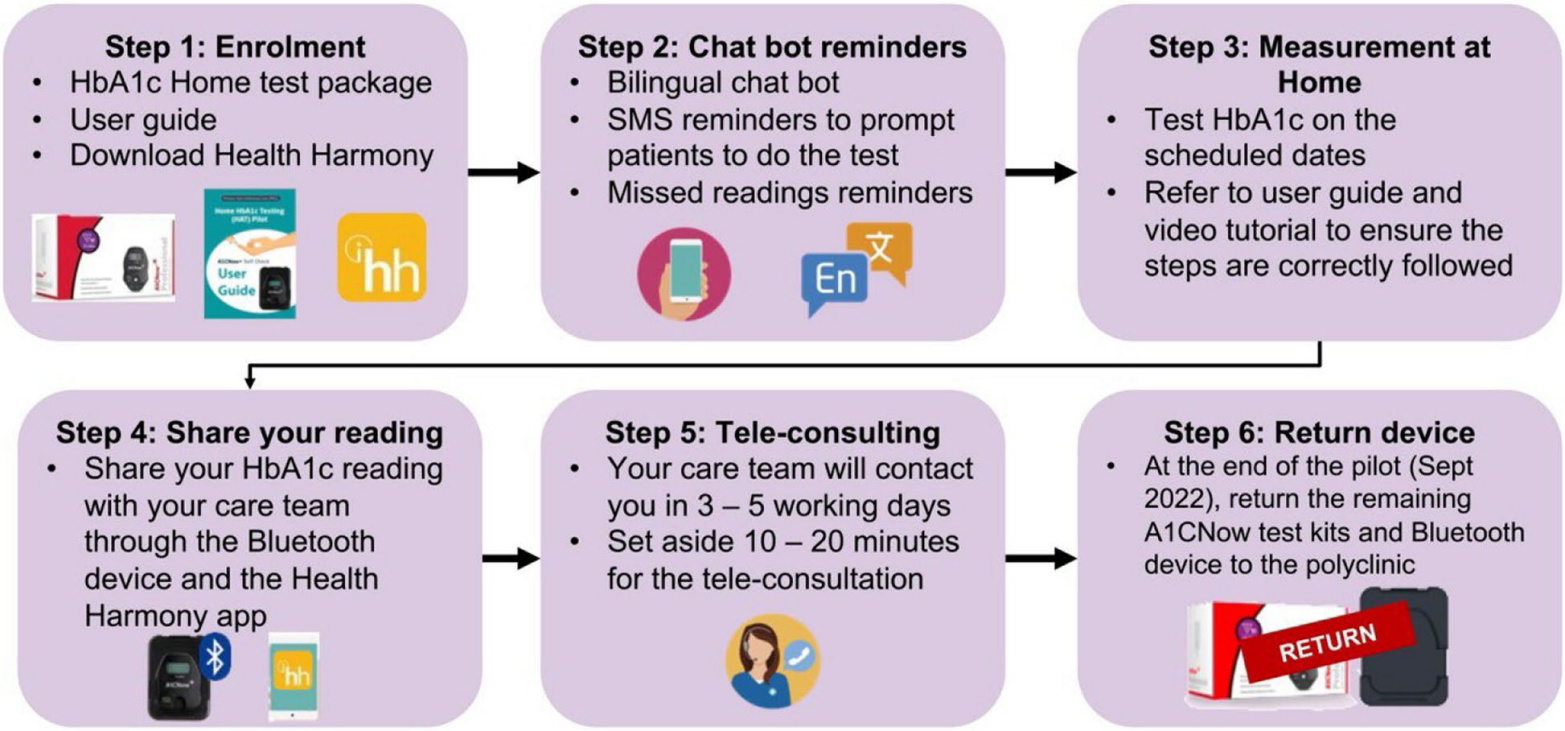
PTEC HAT care plan

**Fig. 3:**
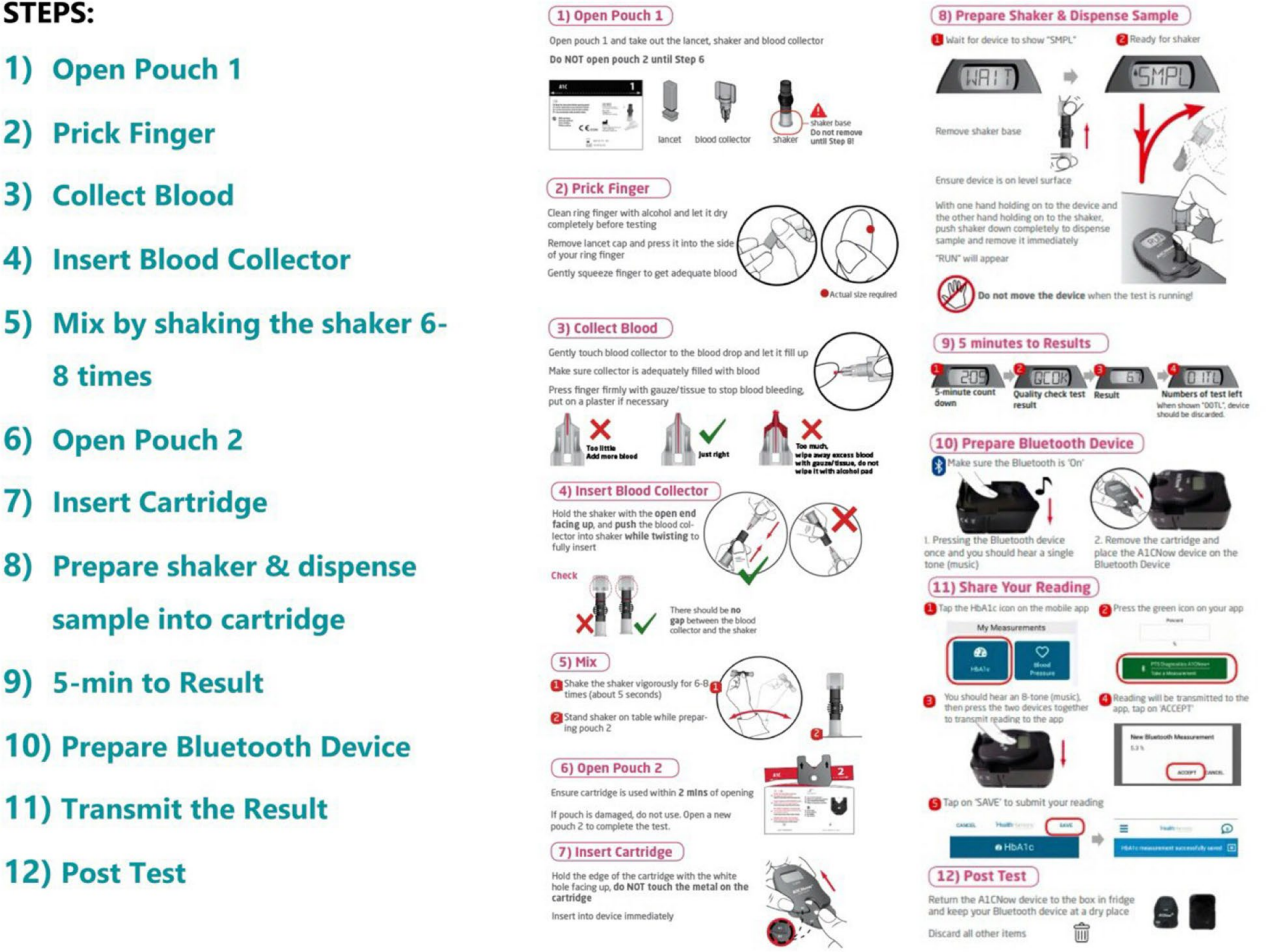
Steps for Home HbA1c Test and Result Submission

The PTEC HAT care plan is illustrated in Fig. 2. Upon enrolment, the patients will be trained by the healthcare team to perform home HbA1c tests through in-person demonstration. They will be issued a Bluetooth enabled HbA1c test kit and other resources, namely, the video tutorial and guidebook to bring home. The test kits require refrigerator storage. When it is due to perform the home HbA1c test, the patients will receive an in-app chatbot reminder notification on their mobile app. Patients will need to thaw the HbA1c test kit reagent an hour before the actual test. The patients are required to follow a 12-step procedure to complete the home HbA1c test, as illustrated in Fig. 3. During the result transmission step (Step 11), the accompanying Bluetooth device will synchronise the reading to the mobile app for the patients to keep track of their HbA1c reading from home. The result will also be transmitted to the healthcare team at NHG Polyclinics for telemonitoring. Patients will then be followed up via teleconsultation. Timely medical interventions will be provided for any abnormal HbA1c reading. The patients will subsequently be reminded to perform home HbA1c testing and submit their readings at regular intervals, until their next scheduled appointment for annual diabetes panel tests and face-to-face consultation at the polyclinic.

This qualitative study is conducted as part of the evaluation of the pilot implementation of PTEC HAT programme. At the time of conducting this qualitative research, the PTEC HAT programme were the first to embed home HbA1c testing into telehealth for low-risk patients with T2DM. It aims to explore the experiences of low-risk patients with T2DM who completed PTEC HAT programme, and to identify the enablers and barriers of using various PTEC HAT components.

## Methods

### Setting

Following the roll-out of PTEC Home BP Monitoring programme [21], the PTEC HAT Tech system, which employed similar technological supports, was piloted in Ang Mo Kio Polyclinic between July 2021 and September 2022.

### Theoretical framework

This study was guided by the non-adoption, abandonment, scale-up, spread, and sustainability (NASSS) theoretical framework [22] in the development of the interview guide and deductive coding. The NASSS framework was developed to study unfolding technology programmes in real-time and particularly to identify and manage their emergent uncertainties and interdependencies [22]. It is one of the most comprehensive framework to predict the success of technology-supported health and social care programmes, and consists of evaluating the challenges across seven domains: 1) the condition, 2) the technology, 3) the value proposition, 4) the adopter system including staff, patient, and caregiver(s), 5) the healthcare organisation(s) including attention to the work of implementation and adaptation, 6) the wider context (institutional and societal), and last, 7) the interaction between domains and adaptation of technology programme over time.

### Sampling and recruitment process

The inclusion criteria for PTEC HAT were: (a) age: 21—80 years old, (b) T2DM patients with HbA1c ≤ 8% (c) no diabetes-related complication such as nephropathy or macrovascular diseases that require more than the usual three to four visits to NHG polyclinic per year, (d) smartphone is supported by PTEC HAT. The pilot implementation of PTEC HAT excluded patients (a) with cognitive impairment, (b) who were pregnant, (c) with pre-existing anaemia of any cause, (d) with a history of ischaemic heart disease, congestive heart failure, stroke, transient ischaemic attack, atrial fibrillation and renal impairment, (e) with complications or target organ damage or complex medical conditions e.g. Parkinson’s disease, dementia, etc., (f) who were on active titration of medication such as angiotensin converting enzyme inhibitors/angiotensin-receptor blockers, and (g) who were concurrently participating in another clinical study or programme involving a novel therapeutic drug/device, at any time during the study period.

Thirty three patients who participated in PTEC HAT programme, with successful transmission of their home HbA1c reading and received teleconsultation, were then referred by their primary healthcare team to the study team. The patient flow chart is detailed in another published [23] article. The study team then contacted the patients for their interest and availability to participate in a semi-structured individual in-depth interview to share their experiences on PTEC HAT programme. Eligible patients were purposively sampled to achieve variation in age groups, ethnicity and gender. The principal investigator, coinvestigators or trained study team members then confirmed patients' eligibility, explained the study and obtained written informed consent. Sufficient time was provided to the patients to consider participation and the voluntary nature of the research participation was emphasised. Informed consent was obtained from the patient before the start of any research procedures.

### Data collection

Data were collected in the form of individual in-depth interviews between April and July 2022 remotely through Zoom video conference. The patients were advised to find a private room for the virtual interview. The interviews took place via Zoom video conference and lasted between 60 and 90 min. Patients’ sociodemographic information, diabetes and medication history were collected at the start of the interview via an interviewer-administered questionnaire (See additional file 1—Questionnaire). This was followed by a semi-structured interview conducted by the principal investigator and observed by study team member(s).

An interview guide was designed with reference to literature and discussion among the research team members. Please refer to Table 1 for more details.

**Table 1:** Interview guide

Domain	Sample question
Experience of learning to do home HbA1c test	1. Please share your experience of learning and using the self HbA1c test 2. What do you think of the coaching done by the care manager? 3. What do you think of the materials and guidebook prepared? How helpful are they? 4. [Probe] How do you think this PTEC HAT programme is different from glucose monitoring done at home? 5. Please can you elaborate more on what you’ve mentioned…? (Probing question) 6. That is interesting. Please tell me more about…. (Whenever there is new data/theme arise)
Experience of the technology (smartphone HealthHarmony Apps & in-app chatbot)	1. How is your experience of learning and using the PTEC HAT smartphone HealthHarmony app? 2. How do you find the interactions with in-app chatbot under the PTEC HAT pilot programme? 3. (Probing question) Do you have any concern if you need to interact and reply to the in-app chatbot using SMS service? 4. Please share your experience with using the smartphone HealthHarmony Apps to send HbA1c results to the care team? 5. Please elaborate more on what you’ve mentioned…? (Probing question) 6. That is interesting. Please tell me more about…. (Whenever there is new data/theme arise)
Experience of using the HbA1c test kit at home	1. One of the key features of PTEC HAT is to perform the HbA1c test remotely, can you share your experience with me? 2. Can you share your experience with storing and preparing the HbA1c test kit? 3. What is the result of your recent home HbA1c test? 4. Do you have any concerns about performing the HbA1c test yourself at home? What are they? 5. What challenges do you face when performing the HbA1c test at home? 6. Please can you elaborate more on what you’ve mentioned…? (Probing question) 7. Do you have any issue in pricking your finger to collect blood samples? 8. Do you encounter problems in mixing your blood sample into the blood collector? 9. Do you have an issue with inserting the cartridge into the HbA1c device? 10. Do you have any issue in getting HbA1c result on the HbA1c device? 11. Do you find any issue in docking the HbA1c device into the Bluetooth dock? 12. Do you encounter any issue in transmitting HbA1c reading from the Bluetooth dock to the HealthHarmony App on smartphone? 13. I find it interesting. Can you tell me more about…? (Whenever there is new data/theme arise)
The role of family/social support in PTEC HAT adoption and experience	1. How would you rate the importance of family/social support in your participation in the PTEC HAT programme? 2. How has family/social support affected your participation in the PTEC HAT programme? 3. What kind of support have you received from your family members or friends in the PTEC HAT programme? 4. What kind of support would you like to receive from your family/friends/healthcare team in the PTEC HAT programme?
Experience of teleconsultation	1. Have you received teleconsultation by the care team? 2. How do you find interacting through teleconsultation with the care team?
Broad experience of PTEC HAT programme	1. Please share with me why you choose to be enrolled? 2. In general, how is your experience of the PTEC HAT pilot programme? 3. In your opinion, what type of person would likely take up the PTEC HAT programme? 4. [Probe] Do you share your diabetes condition with anyone? *(ask about if they are worried about people knowing their diabetes diagnosis)?* 5. Please can you elaborate more on what you’ve mentioned…? (Probing question) 6. That is interesting. Please tell me more about…. (Whenever there is new data/theme arise)
Overall thoughts about PTEC HAT	1. How has PTEC HAT impacted the way you manage diabetes? 2. Please share your suggestions or recommendations on how the program can be improved? What do you think of PTEC HAT vs routine diabetes care by NHGP?
Scalability, Spread & Sustainability	1. How confident are you with the HbA1c self-test result? 2. How likely are you going to continue the HbA1c self-test if PTEC HAT becomes a chargeable service? 3. How much will you expect yourself to pay for this programme? 4. Will you continue PTEC HAT if it is Medisave deductible?
Final remarks	1. Do you have anything else you would like to share with me?

The interviews followed the natural progression of a conversation and the questions were not necessarily covered in the stated order. The patients were given the opportunity to discuss freely based on the questions asked. Probes and follow-up questions were used throughout the interviews to facilitate discussion. The interview questions were modified over the course of the study, using an iterative process that was informed by the content of previous interviews. Each patient received an honorarium at the end of the interview. The interviews were digitally audio-recorded and transcribed verbatim with written consent from the patients. Deidentified transcripts were then used for analysis. Relevant field notes and observations were captured after each interview. The key findings were summarised and added into a Rapid Research Evaluation and Appraisal Lab (RREAL) sheet that allowed the study team to synthesise the data and revise the interview guide as the data were being collected [24].

### Data analysis

Each transcript was read repetitively to ensure accurate understanding of the verbatim and the data analysis was conducted independently by the principal investigator and coinvestigator. The accuracy of the transcripts was verified against the recordings. This was performed with data collection simultaneously to enable the sorting of data into categories [25]. The investigators recognised that their clinical background and preconceptions could influence data collection and interpretation. As such, reflexivity was employed to ensure minimal bias on the data collection and analysis process. Thematic analysis was used for analysis of the data. Initial open coding was carried out using reflexive iteration. The study team regularly met up to discuss the initial codes and the differences in opinions were resolved through consensus. The enablers and barriers identified in the form of rich descriptions were mapped to the emerged themes.

NVivo 12 (QSR International Pty Ltd) was used for data analysis. An excel spreadsheet was used separately by the coinvestigator, which involved generating a matrix to chart the data with the cases in rows, codes in columns and to summarise data in the cells.

This qualitative study was approved by the Institutional Ethics Board (Ref No. 2021/01075).

## Results

**Fig. 4:**
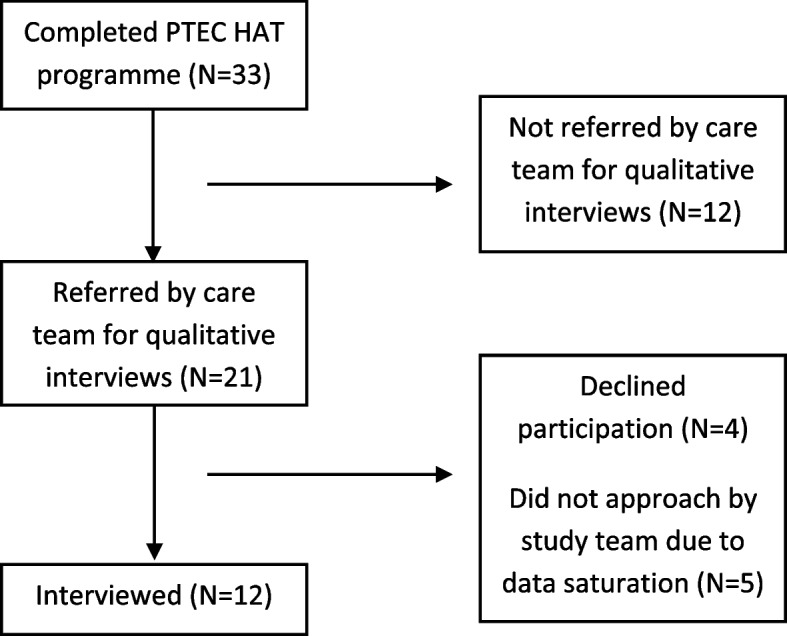
Patient flow chart

Twenty one of the 33 patients who completed PTEC HAT programme were referred by the care team to the study. Four patients declined to be interviewed when contacted by the study team. A total of 12 patients were interviewed (refer to Fig. 4). Interviews were not conducted for the rest of the five patients after attaining data saturation after coding for the 10^th^ interview. The 11^th^ and 12^th^ interview we done to further confirmed data saturation. Ten of the interviews were conducted in English, while the remaining two were in Mandarin. Interviews administered in Mandarin were transcribed verbatim and translated into English.

Twelve patients aged 44 to 75 years were interviewed. Female made up 58.3% (*n =* 7) of the patients interviewed. The baseline characteristics of patients is shown in Table 2. Among them, 83.3% (*n =* 10) were Chinese, 75% (*n =* 9) attained preuniversity and above education, 58.2% (*n =* 7) were working full-time, 66.7% (*n =* 8) had duration of T2DM of not more than five years, 75% (*n =* 9) were on diabetes medication and 83.3% (*n =* 10) had prior experience with using health monitoring devices.

**Table 2:** Baseline characteristics of patients

Baseline characteristics	Number (%)
N	12
Gender	
Male	5 (41.7))
Female	7 (58.3)
Age group at interview (years)	
40–49	2 (16.7)
50–59	5 (41.7)
60–69	5 (41.7)
Ethnicity	
Chinese	10 (83.3)
Indian	1(8.3)
Others	1 (8.3)
Educational level	
‘N’ Level/’O’ Level/NTC and below	3 (25.0)
‘A’ Level/Diploma	4 (33.3)
University	5 (41.7)
Marital status	
Single	2 (16.7)
Married	9 (75.0)
Divorced/Separated	1 (8.3)
Employment status	
Full-time	7 (58.3)
Part-time	2 (16.7)
Unemployed & not studying	3 (25.0)
Diabetes duration	
0–5 years	8 (66.7)
6–10 years	3 (25.0)
> 10 years	1 (8.3)
On diabetes medication	
Yes	9 (75.0)
No	3 (25.0)
Health monitoring experience	
Yes	10 (83.3)
No	2 (16.7)
HbA1c (at baseline)	
< 7%	6 (50.0)
7–7.9%	6 (50.0)

The key findings of this study were reported in the form of rich descriptions and broadly grouped into 5 themes, which are 1) patients’ experiences on PTEC HAT, 2) remote monitoring of T2DM—patient perception, 3) technology component of PTEC HAT—patient perception, 4) value proposition of the PTEC HAT and 5) patient factor in adoption of the PTEC HAT. The findings under each theme were subcategorised into the enablers and barriers.

### The patients’ experiences

#### Enablers

##### Coaching

*‘They first demo to me how to use the set and it’s quite intuitive because it’s a one-to-one training, which is good, so at any point in time if I have a question I can always ask la.’ (Interviewee 2, Female, 52 years old)*

##### User guide and video tutorial

*‘I was quite relieved because there was an instruction pamphlet that I could follow. When I opened the mobile app, there was actually a portion that I could click for the video.’ (Interviewee 12, Female, 51 years old)*

*‘…the video basically refreshes everything you’ve done at the clinic during the demonstration’ (Interviewee 7, Male, 58 years old*

#### Barriers

##### Prolonged onboarding

*‘They didn’t really prep me that the whole learning process was quite long. Actually I remember it take about more than an hour’ (Interviewee 12, Female, 51 years old)*

##### Storage and preparation of the HbA1c testing kit

*‘Packaging too big. Bulky.. so my wife was saying you are taking space in my fridge’ (Interviewee 7, Male, 58 years old)*

*‘Afraid if there’s power outage, afraid this (test kits) will spoil’ (Interviewee 1, Female, 61 years old)*

*‘That is a bit troublesome ah, I need to wait 1 h, then during that 1 h probably you are doing some stuff then after that you forgot about it. Yeah, so I don’t know whether it will affect the reading after you actually left it to thaw for more than an hour. (Interviewee 3, Female, 44 years old)*

### Remote monitoring of T2DM—patient perception

#### Enablers

The patients accepted that the low-risk T2DM is a suitable medical condition for home HbA1c testing, telemonitoring and follow-up through PTEC HAT programme. Patients also shared that there is potential in empowering patients for self-monitoring.

*‘I believe if someone who doesn’t prick and test the blood sugar, it will be revelational for them. You can actually test and see your sugar level yourself. And then for someone who doesn’t do it they can also build their confidence that actually they should consider doing a regular (HbA1c) test.’ (Interviewee 2, Female, 52 years old)*

During the COVID-19 pandemic, PTEC HAT was advantageous in ensuring continued diabetes care.

*‘PTEC HAT is really good in a situation now, because of the COVID, yeah, so if the clinic gets overcrowded, the chances of people getting infected from one another is higher, so this place is an added advantage.’ (Interviewee 9, Female, 60 years old)*

#### Barriers

Many patients expressed that the interval between onboarding and performing the home HbA1c test was lengthy. The HbA1c test, unlike the routine home blood glucose test, was typically performed at three to six monthly intervals to assess diabetes control. This made testing a challenge as the steps for home HbA1c testing becomes less familiar to patients a few months after enrolment.

*‘We can understand very well at that point of time. But… there is a lapse of time. When we do the actual one 6 months later…I can confirm that we will not do it well.’ (Interviewee 8, Male, 63 years old)*

### Technology component of PTEC HAT—patient perception

Overall, the patients valued the technology that comes with the mobile app. They found the in-app chatbot reminder particularly useful when preparing for the home HbA1c testing. However, there were differing views about the home HbA1c testing process and the subsequent teleconsultation. Many patients spoke negatively about the data transmission via the Bluetooth device provided in the package.

#### Enablers

Patients found the in-app chatbot very helpful in reminding them to perform the home HbA1c testing on the prearranged date. In addition, patients expressed that the instructions sent out by in-app chatbot provided assurance for them to complete the home testing correctly.

***‘*** *I think the reminder is good because uh for people who are busy like me…it helps to remind me on that day I am supposed to do this test I have to make sure I keep my time for the appointment.’ (Interviewee 9, Female, 60 years old)*

*‘… (the in-app chatbot) reminds you that on this day you need to do the test, remember to take out and thaw 1 h before using it. So it is a good reminder… (It is) also an add-on to tell me that I'm doing the right thing correctly…’ (Interviewee 4, Female, 45)*

#### Barriers

##### Home HbA1c result transmission with bluetooth device

The Bluetooth device synchronises the HbA1c result from home test kit to the mobile app, thereby minimising transcribing errors by the patients. The Bluetooth device functions by pressing on the unit to turn on and subsequently two distinctive tones are played to indicate that the device is ready to be used and after it has been successfully paired with a smartphone. However, there are no visual cues.

A large majority of the patients in this qualitative study encountered failure when using the Bluetooth device to synchronise home HbA1c reading from the home testing to mobile app, which resulted in negative experience and dropped in confidence.

*‘The part of transmitting result is confusing… I don’t know what does the tone sound like, so the first transmission didn’t get through. It’s a negative feeling because I’ve already pricked my finger and have the HbA1c reading, then the transmission is kind of failed… The confidence level is affected… result in certain level of stress.’ (Interviewee 2, Female, 52 years old)*

*‘…the final procedure is to send the data over, but since you cannot send the data over, then what you have done in front is all wasted’ (Interviewee 8, Male, 63 years old)*

### Value proposition of PTEC HAT

Patients valued the convenience of performing the HbA1c testing at home but the perceived value depended on their employment status.

#### Enablers

The instant result generated by home HbA1c testing was rewarding. It increased the self-efficacy of the patients in improving diabetes control.

*‘I will also like to see results immediately, I don’t have to wait for doctor to tell me or whoever to tell me after waiting for another 45 min at the clinic’ (Interviewee 7, Male, 58 years old)*

PTEC HAT pilot programme was found to be convenient and time-saving as it was able to reduce the number of visits to the polyclinic.

*‘…I will take leave to go for medical appointment and I think it is very troublesome. So if let’s say all these could be done at home, I think it will be very useful not only for COVID but actually for …scheduling of my work…that is why I actually thought it is very good.’ (Interviewee 6, Male, 52 years old)*

*‘…I think this teleconsultation…is fine…is really saving time going down to see a doctor.’ (Interviewee 3, Female, 44 years old)*

The flexible test date or time had additional value in ensuring adherence to perform home HbA1c testing among the patients.

*‘…that’s why I prefer to continue this, Because I can… decide when I can do it: this morning, or maybe this evening or tomorrow morning’ (Interviewee 6, Male, 52 years old)*

Patients felt at ease having the teleconsultation as the healthcare team who contacted the patient was well-informed of their medical condition.

*‘So the standard (of teleconsultation), I'll say is maintained…Experience is the same (as physical consultation).’ (Interviewee 2, Female, 52 years old)*

*‘She seems to know what is happening, so I feel comfortable. Not only like now we are quite used to remote (consultation), but she (also) knows what she is talking about’ (Interviewee 6, Male, 52 years old).*

#### Barriers

The perceived time saved from the programme was diminished for patients with other medical conditions, which also required other tests in the clinic.

*‘It doesn’t take away my time or it doesn’t save me a lot of time just because I am doing this at home.’ (Interviewee 7, Male, 58 years old)*

Some patients found the home HbA1c testing complex and were concerned if the programme became a chargeable service in the future.

*'I think these items are expensive…It is not like that the (glucometer) strip where you test and you don't have a proper reading you can just throw away the strip and just do another prick test…because of the number of steps it requires, every step is important otherwise there is failure of the data (generation and/or transmission). So if the patient is paying for these then it’s also the concern of the patient to do it right the first time. Right?’ (Interviewee 7, Male, 58 years old)*

Additionally, the perceived value of PTEC HAT was dependant on the potential cost of the programme.

*‘I'm hoping that maybe it (PTEC HAT) will be a bit cheaper than the actual lab test because…if the prices are the same, or if this one is a bit higher than the lab test, then they might as well go to the lab test, because people (phlebotomist) will do it for them.’ (Interviewee 12, Female, 51 years old).*

A patient did not think the teleconsultation was helpful but instead, saw it as a standard operating procedure (SOP) and viewed the teleconsultation to be inadequate compared to a physical consult. However, the patient was not aware of the difference between the readings obtained from SBGM and HbA1c test kit as well as the requirement to consult healthcare team in clinic polyclinic visit with every routine HbA1c testing. This could have resulted in the lower perceived value of teleconsultation.

*‘…this telecommunication* *,* *teleconversation, probably is just the SOP, I think that this does not help much’ (Interviewee 8, Male, 63 years old)*

*‘Anyone who has medical issue will still want to seek professional advice, attention, you’ll still want to see a doctor probably, face-to-face…that is where you have more peace of mind’ (Interviewee 3, Female, 44 years old)*

### Patient factors in adopting PTEC HAT

The successful PTEC HAT participation provided a snapshot of the potential take-up rate if it was to become an official programme.

#### Enablers

It was found that a reasonable level of digital literacy, prior experience with blood pressure and glucometer monitoring, absence of needle-related distress, and strong intrinsic motivation for self-monitoring were identified as the main factors attributing to successful PTEC HAT participation.

##### Digital literacy

*‘I'm a smartphone user so l'm able to understand… what to do, so it's after I download right…they ask me to key in some administrative stuff, and everything is I done it on the spot’ (Interviewee 4, Female, 45)*

##### The absence of needle-related distress

*‘I actually have no issue pricking my fingers because first of all, I've been using the glucometer myself at home as well also, so that requires a lot of pricking. …when they told me oh, you still need to actually prick your fingers, I said it's not a problem.’ (Interviewee 3, Female, 44 years old)*

##### Intrinsic motivation and prior experience with health monitoring

*‘I came to a point where I'm really concerned about my wellbeing… That I might have hypertension, so I thought this is a good way for me, checking on myself…that's why I signed up for it…there is a big correlation because people who are interested in their results then they will buy the glucometer and prick.’ (Interviewee 11, Male, 62 years old)*

#### Barriers

Conversely, patients with lower level of digital literacy, needle-related distress and low level of self-motivation would have a lower likelihood of overcoming challenges when interacting with the multiple components of PTEC HAT.

##### The inadequate level of digital literacy

*‘….my wife (is) bad with technology…my sister-in-law was in my house and I purposely take out these kits, ask her for help…she is more educated on technology and yet we follow all the SOP, (we) cannot get through’ (Interviewee 8, Male, 63 years old)*

##### Needle-related distress

*‘I'm scared of needles already. I don’t want to prick myself again’ (when the patient failed to transmit the result via Bluetooth)’ (Interviewee 12, Female, 51 years old)*

##### Lack of self-motivation

*‘I don’t have much feelings about it, it’s just that I didn’t know how to at the start and then I thought I didn’t want to use it anymore’ (Interviewee 5, Female, 54 years old)*

## Discussion

This study aimed to explore the experiences of low-risk patients with T2DM who participated in PTEC HAT programme, and to identify the enablers and barriers of using various PTEC HAT components. The suitable patient characteristics enabling the participation in PTEC HAT included reasonable level of digital literacy, health monitoring experience in the past, absence of needle-related distress and strong intrinsic motivation. The key enablers identified for using various PTEC HAT components included the positive reinforcement with instant HbA1c generation, great value of flexibility and convenience, benefits of teleconsultation to reduce polyclinic visit and the perceived suitability low-risk T2DM for home HbA1c testing and telemonitoring. The enablers identified to maintain patients’ motivation in completing the PTEC HAT programme included the coaching by primary care team, the support in the form of in-app chatbot reminder, as well as the user guide and video tutorial accessibility. Even though they did not broadly affect patients’ experiences, there were several barriers identified, which included the long interval between onboarding and subsequent home HbA1c testing, making challenging for patients to recall the steps, persistent issue with HbA1c result transmission with Bluetooth device, perceived inferiority of teleconsultation, concern of potential cost of the programme and the amount of efforts needed to perform self-testing as well as unsuitable patient characteristic—low digital literacy, needle-related distress and lack of motivation.

### The patients’ experiences

Sander et al. found that high level of uncertainty regarding the technological aspects of the health device being offered was one of the reasons for non-adoption [26]. For PTEC HAT, patients shared that there were several initiatives established to minimise the uncertainty, which enhanced this learning experience during the onboarding session. The engagement, availability of training material and support delivered during the in-person coaching provided assurance to the patients the effectiveness of the technology. The return demonstration performed in the clinic gave the patients confidence that they can repeat the testing at home without the physical assistance from the care team. The additional resources provided to them in the form of video tutorial and the user guide served as mitigating factors to perform the HbA1c testing even after three to six months post training.

#### The enablers

Our findings revealed that the perceived good value by the patients as the key facilitator for completing PTEC HAT programme. Majority of the patients recognised that telehealth, with technology enhanced home HbA1c testing, as a convenient alternative for the routine care in the clinic. This perceived value was dependent on the compulsion for a more flexible schedule that is associated with the patients’ occupation and the amount of free time they have. Most patients do not mind travelling down to the clinic for the regular blood test as they have other appointments to fulfil, such as vaccination, diabetic retinal photography and diabetic foot screening. However, the fact that the PTEC HAT home test is not bounded by time or place made it an attractive alternative to many who have limited free time. This is consistent with the finding of a cross sectional validation study, in which the shortening of travel and waiting times to the clinic was identified as one of the benefits of diabetes telehealth [27].

Another enabler that increased the acceptability is the patients’ prior experience with health monitoring like SBGM, which requires similar technical competency by the patient in obtaining the blood sample, followed by the need to feed the sample into a reader to obtain the glucose reading. A scoping review of 29 studies found that previous experience with the technology is one of the patient-level facilitators for the uptake of digital health technology [28]. Majority of the patients in our study were found to have prior experience with the health monitoring, thus making them a more empowered and motivated group.

#### The barriers

Several patients requested to remove redundant technological features that add complexity to the whole innovation in order to improve the practical acceptability. The primary barrier that the patients faced with the technology was with the result transmission using the Bluetooth device prescribed to them. Our finding was consistent with how Torbjørnsen et al*.,* described the usability issue of Bluetooth device hindering practical acceptability of a telecare programme [29]. Torbjørnsen’s et al*.*’s study was primarily to understand factors affecting a patient’s acceptability of a mobile app for diabetes self-management. It was found that one of the most valued features of the app was its ability to transfer the blood glucose data to health-care personnel using Bluetooth. However, it was also found to be one of the major usability issues, requiring frequent support to reconnect the devices, making it challenging to use. The need to send the HbA1c reading via Bluetooth device was seen as a redundant feature that resulted in negative emotions and reduced the usability of the technology. The patient’s perceived barriers echoed the findings from other studies exploring the factors impeding the acceptance of digital health technologies, namely, poor design and inoperability of the telehealth programme [30].

Some of the patients were not aware of the fundamental difference between the readings obtained from SBGM and HbA1c test kit. This could have resulted in the lower perceived value of PTEC HAT programme and the lack of appreciation for the additional steps required by PTEC HAT to obtain the reading. This highlighted the importance of better patient education to illuminate the significance and value of HbA1c in self-management of T2DM.

The low frequency of HbA1c testing was shared by the patients as another potential barrier in joining the programme. They did not see the cost-effectiveness of investing both time and money in a potentially high-cost device, that requires a certain level of technical and digital competency to operate, which will only be used two to four times a year. Therefore, these factors, as opposed to the physical visit to the clinic that provides a no-hassle-service, makes PTEC HAT less attractive to the patients. In this regard, the growth of value-based payment models may be a solution to provide incentives in implementing cost-effective, high-quality and coordinated telehealth [31].

### Study strengths and limitations

To our knowledge, this is the first qualitative study on low risk adult patients adopting telehealth with technology-enhanced home HbA1c test for T2DM care. The purposive sampling enabled detailed data collection from patients with various demographic profiles. The in-depth individual interviews performed until data saturation enabled comprehensive identification of interweaving enablers and barriers. Since the programme does not require any financial commitment during the pilot phase, patient enrolment may be influenced by the absence of cost, making it difficult to determine actual demand or willingness to pay in a real-world implementation.

The strengths of the study were enhanced by striving to achieve credibility, transferability, dependability and confirmability using Lincoln and Guba framework [32]. A few strategies were used to achieve credibility: 1) investigators maintained prolonged engagement and member-checking was carried out pro-actively during interviews and 2) investigator triangulation was carried out during the process of data analysis. In addressing transferability, the coded data was presented as thick descriptions so that the patient’s context, behaviour and experience are meaningful to the reader. To maintain dependability, records of the conduct of the study and all meetings were documented, thereby establishing a clear audit trail. Confirmability was achieved by including patients’ direct quotes in the results and reference to the literature that confirmed the interpretations in this study.

This qualitative study was limited by the fact that 83.3% of the patients were Chinese, and there was no Malay patient. Therefore, this ethnicity was unrepresented. The small number of patients in PTEC HAT programme (*n =* 33) had also restricted the heterogeneity for purposive sampling. In addition to that, other NASSS domains such as ‘the organisation’, ‘the wider context’ and ‘the interaction between domains and adaptation over time’ were not addressed as the study team did not ask the related views in this study. The transferability of our findings to other groups of diabetes patients may be limited as this study was conducted with low-risk patients with T2DM. Future studies should focus on exploring the perspectives of the healthcare team and other stakeholders in PTEC HAT programme, in terms of the factors associated with non-adoption and the cost-effectiveness of PTEC HAT in diabetes management.

## Conclusion

As telehealth increasingly becomes an integral part of healthcare, it is important to understand and learn from the patients’ experiences in the novel technology-enhanced home HbA1c telehealth programme for T2DM care. Based on the patients’ experiences, our qualitative study established that telehealth with technology enhanced home HbA1c testing is a useful alternative to routine care for low-risk patients with T2DM in primary healthcare institutions. It is not only one that is providing patients with the flexibility and convenience but also empowers patients for self-care. However, the benefit is moderated by lack of practical usability of the Bluetooth device for HbA1c result transmission, as well as concerns of possible high cost associated with the technology component for HbA1c testing that is only done a few times a year, which diminishes the value proposition. It is hoped that the knowledge gained from our study can improve the design of telehealth initiatives for patients with T2DM and possibly integrate PTEC HAT seamlessly into existing programmes for other chronic diseases, thereby enhancing the overall benefit for the patient.

## Supplementary Information

Supplementary Material 1.

## Data Availability

The datasets generated and/or analysed during the current study are not publicly available due to confidential nature of the datasets but are available from the corresponding author on reasonable request.

## Abbreviations

BP: Blood Pressure
CGM: Continuous glucose monitoring
COVID: Coronavirus Disease
DSRB: Domain Specific Review Board
HbA1c: Glycated Haemoglobin
MOHT: MOH Office for Healthcare Transformation
NASSS: Non-adoption, Abandonment, Scale-up, Spread, and Sustainability
NHG: National Healthcare Group
PTEC HAT: Primary Technology Enhanced Care Home HbA1c Testing
RREAL: Rapid Research Evaluation and Appraisal Lab
SBGM: Self-Blood Glucose Monitoring
SOP: Standard Operating Procedure
T2DM: Type 2 Diabetes Mellitus
