# Supplementary material for: Understanding the patients’ experience in Primary Technology Enhanced Care Home HbA1c Testing (PTEC HAT) programme—a qualitative study
Source: BMC Prim Care. 2025 Oct 13;26:309. doi: 10.1186/s12875-025-03034-2 (PMC12519817; doi:10.1186/s12875-025-03034-2)
Supplement: Supplementary file 1 — Supplementary Material 1. [file 12875_2025_3034_MOESM1_ESM.pdf]

Study Title: Understanding the patients' experience in Primary Tech-Enhanced Care Home HbA1c Test (PTEC-HAT) pilot programme – A Qualitative Study

**Section A: Study Information**	
1. Study ID	
2. Date of Interview	____ (DD) / ____ (MM) / _______ (YYYY)
3. Name of interviewer	

**Section B: Demographic**	
1. Gender	☐ 1 Male<br>☐ 2 Female
2. Ethnicity	☐ 1 Chinese<br>☐ 2 Malay<br>☐ 3 Indian<br>☐ 4 Others, please specify: ____________________
3. Year of Birth	
4. Marital Status	☐ 1 Single<br>☐ 2 Married<br>☐ 3 Divorced/Separated<br>☐ 4 Widowed<br>☐ 5 Others, specify: ______________________
5. Highest level of education attained	☐ 1 No formal education<br>☐ 2 Primary<br>☐ 3 Secondary<br>☐ 4 'N' Level / 'O' Level or NTC 3 certificate or its equivalent<br>☐ 5 'A' Level or NTC 1-2 or Certificate in office / business skills or its equivalent<br>☐ 6 Polytechnic diploma, Other diploma & professional qualification<br>☐ 7 University & above<br>☐ 8 Others, please specify: ______________
6. Employment	☐ 1 Employed. Part time / Full time (*Please circle appropriately*).<br>Occupation:_____________________________<br>☐ 2 Studying, specify institution: _______________<br>☐ 3 Unemployed and not studying<br>☐ 4 National Service (NS)
7. Type of dwelling	☐ 1 HDB 1-Room Flat HDB<br>☐ 2 HDB 2-Room Flat HDB<br>☐ 3 HDB 3-Room Flat HDB<br>☐ 4 HDB 4-Room Flat HDB<br>☐ 5 HDB 5-Room Flat HDB

	☐ 6 HDB Executive Flat HDB<br>☐ 7 HUDC Flat (Non-Privatized)<br>☐ 8 HDB Studio Apartment<br>☐ 9 Landed Properties / Bungalow / Detached House / Semi-Detached House/ Terrace House<br>☐ 10 Condominiums / Other Apartments<br>☐ 11 Others, please specify:_____________________

**Section C: Medical conditions**	
8. Year of diagnosis and lastest clinic HbA1c	
9. Before joining PTEC-HAT, do you check your blood glucose at home with glucometer?	☐1 Yes<br>☐ 2 No
10. Are you on medication?	☐ 1 Yes<br>☐ 2 No
11. Date of enrolment into PTEC-HAT	
